# Antinuclear Antibodies With a Homogeneous and Speckled Immunofluorescence Pattern Are Associated With Lack of Cancer While Those With a Nucleolar Pattern With the Presence of Cancer

**DOI:** 10.3389/fmed.2020.00165

**Published:** 2020-04-30

**Authors:** Amandine Gauderon, Pascale Roux-Lombard, David Spoerl

**Affiliations:** ^1^Division of Immunology and Allergy, Department of Medical Specialties, University Hospital and Faculty of Medicine, Geneva, Switzerland; ^2^Division of Laboratory Medicine, Department of Diagnostic, Geneva University Hospitals, Geneva, Switzerland

**Keywords:** neoplasm, immune system, autoimmune diseases, immunologic tests, prognosis

## Abstract

**Background:** Different antinuclear antibody (ANA) patterns have been associated with the presence of cancer, while other are typically seen in autoimmune diseases. This study aims to investigate the association between ANA and cancer, focusing on patients with ANA with a nucleolar indirect immunofluorescence (IIF) pattern.

**Materials and Methods:** ANA patterns and positivity of antibodies against nuclear antigens (NA), in particular those responsible for a nucleolar ANA pattern and/or associated with systemic sclerosis (CENP-A/B, fibrillarin, Ku, NOR-90, PM/Scl-100, PM/Scl-75, RNAP-III, Scl-70, Ro52/TRIM21, and Th/To) were analyzed and correlated to an internal database of patients with cancer.

**Results:** The study included 15,728 patients who had an ANA analysis, 386 patients who had immunodot analysis for antibody/ies against/to specific NA and 15,701 patients diagnosed with cancer. The presence of ANA with a nucleolar pattern showed an increased relative risk (RR 1.5, 95%CI 1.03-2.3) for an associated cancer. Anti-Scl70 and anti-RNAP-III were associated with cancer in 15 and 14%, respectively. The presence of ANA with a homogeneous & speckled (HS) pattern was significantly associated with the absence of cancer (*p* < 0.01). Patients with a HS pattern were found to have a lower relative risk (RR 0.7, 95%CI 0.5-0.9) of having cancer compared to those with other patterns.

**Conclusions:** Larger studies are needed to investigate which particular antibody/ies against/to specific NA is responsible for the association between nucleolar ANA and cancer, but anti-Scl70 and anti-RNAP-III, which is frequently associated with the presence of anti-RNAP-I, are good candidates to explain this association. Patients with a nucleolar pattern might be considered for cancer screening, in particular if they have anti-Scl70 and anti-RNAP-III antibodies.

## Introduction

Autoantibodies against proteins located in the nucleus are called antinuclear antibodies (ANA). ANA can be detected efficiently and sensitively by indirect immunofluorescence (IIF) when suspecting an autoimmune disease (AID). Antigen specific tests usually follow positive ANA tests to give more information on the specific targeted nuclear antigens (NA) (1).

In IIF, ANA can display different nuclear patterns depending on the targeted antigen. These patterns were recently defined by the International Consensus on ANA Patterns (ICAP) (2). Anti-nucleolar autoantibodies (ANoA) can be homogeneous (AC-8), clumpy (AC-9), or punctate (AC-10). The simultaneous presence of two or more patterns is possible and a frequently observed combination is the homogeneous & speckled (HS) pattern, later controversially termed as “dense fine speckled,” which is mostly associated with anti-dense fine speckled antigen 70/Lens epithelium-derived growth factor (DFS70/LEDGF) specificity (3). A very similar aspect but without anti-DFS70/LEDGF specificity has recently been termed “pseudo-DFS pattern” (4).

In clinical practice, the presence of high titers of ANA raises the suspicion of an underlying AID. Additionally, several studies have shown the association of ANoA, historically linked to SSc and myositis, with malignant disease (5–8). Other studies have shown the presence of anti-RNA polymerase III (anti-RNAP-III) in many different types of cancers, particularly breast, lung and hematological cancers (9–14). Anti–RNAP-III antibodies have been shown to almost unequivocally coexist with anti–RNA polymerase I (anti-RNAP-I) antibodies (15). This complicates the identification of anti-RNAP-I in that its punctate nucleolar staining may be obscured by the coexisting nuclear IIF staining of anti–RNAP-III antibodies (15).

Despite the association of ANA with malignant disease, different studies have shown that patients with ANA had a more favorable cancer outcome when compared to patients negative for ANA, possibly explained by more vigorous activation of the immune system fighting against the malignant cells (16–20).

This retrospective study aims to investigate the relationship between the presence of different ANA patterns, antibody/ies against/to specific NA and the presence or outcome of cancer.

## Materials and Methods

### Patients

Patients tested for ANA by IIF and for antibody/ies against/to specific NA by immunodot in the University Hospital of Geneva's immunology and clinical allergology laboratory were analyzed and compared to a database of all patients who presented at the same center diagnosed with cancer according to the ICD-10 code, between 2010 and 2016. Patients tested for ANA or by immunoblot but not included in the latter database were considered to not have cancer.

Patients who had positive ANA exhibiting different IIF aspects in two separate analyses (for example: first analysis speckled and then nucleolar aspect in another analysis) or have been tested once positive and once negative during this time, were subsequently excluded in order to obtain a cohort without ambiguous IIF pattern.

Immunodot results of 10 specific antigens (CENP-A/B, fibrillarin, Ku, NOR-90, PM/Scl-100, PM/Scl-75, RNAP-III, Scl-70, Ro52/TRIM21, and Th/To), known to possibly elicit a nucleolar ANA pattern or to be positive in SSc, were further analyzed.

For patients with a nucleolar pattern, as well as those with a HS pattern, medical charts were studied in order to confirm the cancer diagnosis and analyze the survival rate. Ethical approval was obtained from swissethics (study no. 2017-02046) in accordance with the World Medical Association's Declaration of Helsinki.

### Antibody Testing

Our laboratory has accreditation by the Swiss Accreditation Service on the basis of the relevant international standards: ISO/CEI 15189. ANA detection was performed by IIF technique using HEp-2 (human epithelial cell line) substrate slides and FITC anti-human IgG conjugate (Nova Lite™ Inova Diagnostics, San Diego, USA). Serums were tittered from 1/80 to endpoint and results were expressed as the last positive dilution. ANA titer ≥1/160 were considered positive. The ICAP classification was gradually introduced in our laboratory only in 2016. The term HS, which still sometimes is seen without an association with anti-DFS70/LEDGF antibodies, was used in our study to describe both the dense fine speckled (AC-2) and other patterns with a speckled nuclear staining associated with a stained metaphase plate.

According to an internal algorithm, antigen specificity was tested in the presence of ANA with nucleolar pattern at a titer ≥1/320 and in patients suspected to have SSc by the treating physician with the EUROLINE SSC Profile (Nucleoli) immunodot (EUROIMMUN AG, Luebeck, Germany) and scanned with CanoScan Lide 100. Antibodies against Scl-70, CENP A, CENP B, RP11 (RNAP-III), RP155 (RNAP-III), fibrillarin, NOR-90, Th/To, PM/Scl-100, PM/Scl-75, Ku, Ro52/TRIM21 were tested and analyzed with EUROLineScan software. Intensity values above 50 were considered positive.

### Statistics

Two statistical approaches were used in order to increase the reliability of the results: First we tested whether the rate of positivity of a particular ANA pattern among patient who had cancer was significantly different from those who did not have cancer, i.e., if there was an association. In a different approach, we tested if the risk to have cancer with a given pattern was significantly different than the risk to have cancer with any other pattern, i.e., the relative risk. Fisher test and two-tailed Chi-square with Yate's correction were used depending on the number of patients. Two-tailed unpaired student t-test was used to compare two means, that is the age and delay in **Table 2**. For age, the Kolmogorov-Smirnov test of normality showed a D value of 0.13, *p*-value was 0.74, indicating likely normal distribution. The Shapiro–Wilk test gave a similar result when excluding outliners. A value of *p* < 0.05 was considered statistically significant. The free available internet version of GraphPad QuickCalcs and MedCalc were used.

## Results

### IIF Results

**Figure 1:**
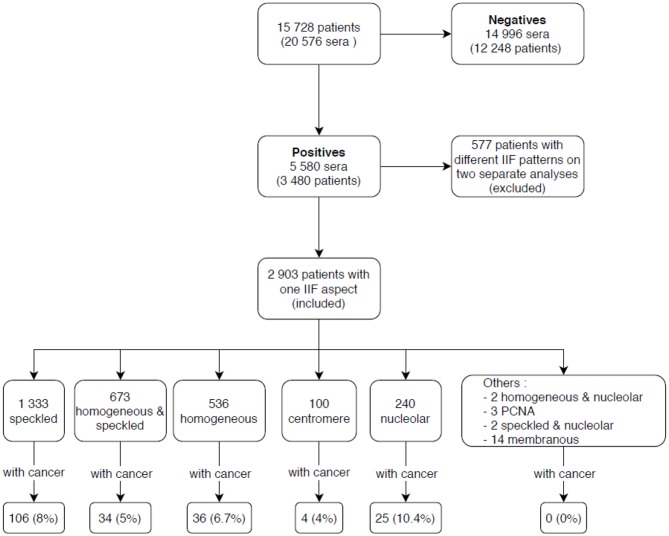
Indirect immunofluorescence (IIF) results of patients included in the study. IIF, indirect immunofluorescence; PCNA, proliferating cell nuclear antigen.

Among 15,728 patients tested by IIF during the study period, 2,903 had ANA titers ≥ 1/160, after the exclusion of 577 patients who had inconsistent duplicate results, as outlined in the methods. Figure 1 shows the various IIF patterns that were observed among the ANA positive patients.

### Cancer Diagnosis

Among 23,195 patients diagnosed with neoplasm, 15,701 patients had malignant disease (463 different ICD-10 diagnosis, block C) and 7,494 patients had either benign disease or *in situ* neoplasms (281 different ICD-10 diagnosis, block D).

### Association Between Malignant Disease and IIF Pattern

Association analysis of each ANA pattern with the presence of malignant disease showed that 10.4% of patients with the nucleolar pattern had malignant disease compared to 8.0% without this pattern, the difference not reaching statistical significance (*p* = 0.2). Similarly, other patterns analyzed (speckled, homogeneous, centromere) were not statistically associated with the presence of malignant disease, except for the HS aspect that was found to be significantly associated with the absence of malignant neoplastic disease (*p* < 0.01).

### Relative Risk

A total of 1,217 patients had both an ANA analysis and a malignant cancer. 205 patients had positive ANA and had a malignant neoplastic disease (Table 1). The HS pattern was found to have significantly a lower relative risk (RR 0.7; *p* = 0.02) compared to patients with other ANA patterns. On the contrary, the nucleolar pattern showed an increased relative risk (RR 1.5; *p* = 0.04).

**Table 1:** Relative risk of malignant disease according to ANA pattern.

**Positives IIF patients**	**Malignant neoplasm (*n* = 1,217)**	**No malignant disease (*n* = 13,934)**	**Relative risk**	**95% CI**
Speckled (*n* = 1,333)	106 (8%)	1,227 (92%)	RR = 1.2	0.9 to 1.6
Homogeneous & speckled (*n* = 673)	34 (5%)	639 (95%)	RR = 0.7	0.5 to 0.9
Homogeneous (*n* = 536)	36 (7%)	500 (93%)	RR = 0.8	0.6 to 1.1
Nucleolar (*n* = 240)	25 (10%)	215 (90%)	RR = 1.5	1.03 to 2.3
Centromere (*n* = 100)	4 (4%)	96 (96%)	RR = 0.5	0.2 to 1.4
Other (*n* = 21)	0 (0%)	14 (100%)	NA	NA
Total (*n* = 2,903)	205	2,698		

*NA, not applicable*.

10.4% of patients who were positive for ANoA had malignant cancers, which was significantly higher (*p* < 0.01) compared to the patients with the HS aspect (5%).

### Immunodot Results

**Figure 2:**
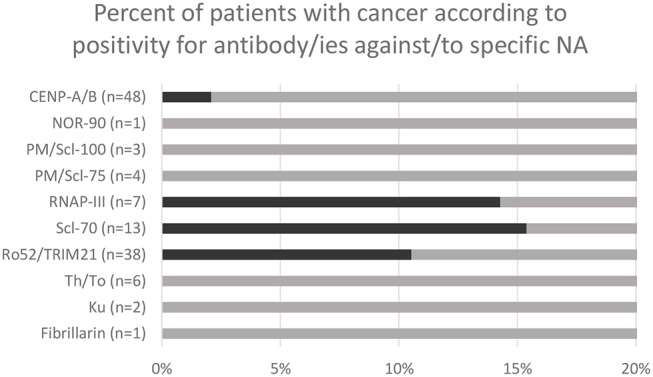
Malignant neoplasm rate in patients with positive immunodot for specific antibody/ies against/to nuclear antigens (NA).

A total of 386 patients have been tested for specific NA over the study period. 123 patients were positive for one or more antibody/ies against/to NA, including 24 patients who were positive for several antigens simultaneously. 48 patients were positive for CENP-A/B, 38 for Ro52/TRIM21, 13 for Scl-70, 7 for RNAP-III, 3 for PM/Scl-100, 4 for PM/Scl-75, 6 for Th/To and 1 for NOR-90, 1 for fibrillarin, 2 for Ku. A total of 8 patients had both antibody/ies against/to NA by immunodot and malignant neoplastic disease (i.e., were found “positive” in both database). No statistical analysis was performed with *n* = 8. However it is worth noting that two (15.4%) of the patients with anti-Scl-70 and 1 (14.3%) of the patients with anti-RNAP-III had malignant disease (Figure 2).

### Clinical Characteristics of Patients With Malignancy and a Nucleolar or a HS Patterns

The medical charts of patients with a HS or nucleolar aspects were analyzed to confirm the diagnosis of malignant disease and investigate the clinical characteristic, including the survival.

The average age of patients with ANoA was 60 years at cancer diagnosis. During the study period, 12 died (48%), at an average age of 66.9 years. Four patients had a concomitant AID or paraneoplastic disease.

For patients with a HS pattern, the average age was 66.7 years at the time of cancer diagnosis. 16 patients died (47%), with an average of 75 years at the time of death, this being statistically higher than for the nucleolar pattern (*p* = 0.02). 10 patients had either an AID or a paraneoplastic disease. Mortality cause was not always available from medical charts, so that the all-cause mortality was assessed and is shown in Table 2.

**Table 2:** Characteristics of patients with malignant disease and positive antinuclear antibodies according to indirect immunofluorescence pattern.

	**Nucleolar(*n* = 25)**	**Homogeneous and Speckled (*n* = 34)**	***p* value**
Mean age at IIF analysis [years (95% CI)]	61.6 (55.3–67.9)	68.5 (64–73)	*p* = 0.09
Mean age at cancer diagnosis [years (95% CI)]	60 (53.7–66.3)	66.7 (61.9–71.4)	*p =* 0.07
Mean age at death [years (95% CI)]	66.9 (57.6–75.3)	75 (68.5–81.4)	*p* = 0.02
Mean delay between cancer diagnosis to death [years (95% CI)]	3.2 (1.7–4.7)	4.5 (1.5–7.5)	*p* = 0.65
AID [n]	4 (16%)	10 (29.4%)	*p* = 0.2
- RA	2	0	
- Granomatosis with polyangeiitis	1	0	
- SLE	0	2	
- SjS	0	1	
- Coeliac disease	0	1	
- DM paraneoplastic	0	1	
- PM paraneoplastic	1	1	
- SSc	0	1	
- Polymyalgia Rheumatica	0	1	
- Horton's Disease	0	1	
- Ulcerative colitis	0	1	

*AID, Autoimmune disease; RA, Rheumatoid arthritis; SLE, Systemic lupus erythematosus; SjS, Sjögren syndrome; DM, Dermatomyositis; PM, Polymyositis; SSc, Systemic sclerosis*.

## Discussion

The recent discoveries on the mechanism of CTLA-4 and PD-1 inhibition led to the development of several drugs that have increased the treatment options for a large number of malignant diseases (21). However, these drugs have often side effects, including the development of autoimmune disease. Whether an immunological work-up is required in order to identify patients at risk to develop these adverse immunological reactions is currently matter of debate. This study investigates the association of different ANA patterns with the presence and outcome of cancer. Our study might help clinicians to understand in which patients the effects of the immune system might be protective or deleterious in the context of an associated neoplastic disease, in view of more patient's tailored treatment protocols in the future. Based on our results, it would be interesting to investigate if patients with ANoA respond better to checkpoint inhibitor therapy than perhaps patients with the HS pattern.

ANA were found to be positive in ~40% of patients with malignant tumors in two previous studies (22, 23). Conversely, Shiel and Jason found a malignant disease in 2.9% of patients with positive ANA (24). In an attempt to find a pathophysiological mechanism linking ANA to cancer, several hypotheses have been formulated in the literature. Recently, Giat et al. demonstrated that this relationship might be bidirectional: cancer could be implicated in the emergence of AID as paraneoplastic phenomenon and conversely, some AID and immunosuppressive treatments are known to increase the risk of cancer (25). Other authors also described the implications of two mutated proteins, showing a nucleolar (AC-8) pattern in IIF when targeted by ANA, in the development of tumors. The first, nucleophosmin, is frequently overexpressed and mutated in human cancer. This protein contributes to oncogenesis through many different mechanisms (26). The second, nucleolin, is commonly overexpressed in cancer cells and influences the cell survival, proliferation and invasion, by acting on different cellular pathways (27). Mutation leading to the expression of altered proteins is one possible mechanism leading to an immune response and it is not excluded that this phenomenon might explain the development of ANA, the latter signaling in this context the development of a malignant disease (28). In this setting, some authors have explored the idea of using ANA as markers for early cancer detection (6, 13). A screening recommendation of aggressive malignancies for anti-RNAP-III positive patients with scleroderma was made in 2017 by Sha et al. (13). Lazzaroni et al. highlighted the frequency of breast cancer in patients with anti-RNAP-III antibodies and therefore suggested its screening in anti-RNAP-III positive patients (10). Our results are less specific for a particular NA, but similarly indicate the association between antibodies targeting nucleolar antigens and cancer and indicate the need of a particular close observation in patients with positive ANoA not having yet been diagnosed with a malignant disease.

Differently than most studies investigating the prevalence of cancer in selected cohorts (7–9, 12, 20, 29, 30), our study investigates the link between different ANA patterns and malignant disease in a non-selected population. Our study shows an increased relative risk for malignant disease in patients with ANoA compared to other ANA patterns, as well as a link between the HS pattern and the absence of cancer. These results show that different ANA patterns might herald the involvement of the immune system, either in protecting from malignant disease or in signaling its presence, as shown in other studies (20, 31).

Interestingly, numerous studies linked specific NA, showing a nucleolar pattern in IIF, to cancer, such as PM/Scl (7, 8, 30), NOR-90, and fibrillarin (6, 32). RNAP-III, which has also be linked to malignant disease (9, 11, 12, 29, 33, 34), has been shown to be very frequently associated with the presence of RNAP-I, which shows notably also a nucleolar pattern (15). This could theoretically explain the association between ANoA and cancer found in our study. Moreover, in our study, the two most common NA in cancer patients were Scl-70 (15.4%) and RNAP-III (14.3%). These results were similar to those of Moinzadeh et al., in a cohort of SSc patients (29). Recently, Scl-70, showing a DNA-topoisomerase-like IIF pattern (AC-29), has been described to elicit a punctate nucleolar pattern (35). It is therefore not excluded that some of the patterns identified as nucleolar might have been caused by the presence of anti-Scl-70. In our study, 1 patient with anti-Scl-70 showed indeed a nucleolar pattern, however the number of patients investigated by both IIF and immunodot was too low to draw any conclusions in this regard.

The second main result of our study is that the HS pattern is significantly associated with the absence of cancer and has a lower relative risk than other ANA patterns. Although this pattern has not been previously described as protective, the study of Blaes et al. support our results. These authors found the presence of ANA as an indicator of a protective immune response in a cohort of 61 patients with non-small cell lung carcinoma (19). In this study, the most frequent ANA patterns were homogeneous (55%) and speckled (25%). Another study in patients with lung cancer found that the presence of ANA targeting different nuclear antigen was associated with a prolonged survival without disease progression, suggesting that they might play a protective role in the progression of cancer (31).

In our study, only the HS pattern was associated with a decreased relative risk of concomitant neoplastic disease. Recent studies have shown that in some cases the HS pattern might be related to ANA targeting the DFS70/LEDGF antigen (3). These antibodies have been shown to be frequently found in an “apparently” healthy population (36). Indeed, in a post hoc analysis, 13 out of 14 (93%) patients with a HS pattern in our study were tested for anti-DFS70/LEDGF antibodies and were found positive. In fact, anti-DFS70/LEDGF analysis was introduced in our laboratory in 2016, and only recently we realized that most of the previously described HS patterns were in reality caused by anti-DFS70/LEDGF antibodies and corresponded to the AC-2 pattern according to international consensus on ANA pattern. Unfortunately, the initial retrospective study design did not allow to investigate whether anti-DFS70/LEDGF were associated with the lack of cancer and no conclusions can be drawn from this limited data set. The role of anti-DFS70/LEDGF antibodies is currently matter of debate (36), however if the association between these antibodies and the absence of cancer will be confirmed in the future, this could explain the decreased relative risk of concomitant neoplastic disease in patients with ANA with the HS pattern found in our study.

In our study, data from medical charts revealed a lower average age at death for ANoA positive patients compared those with the HS pattern. However, this is probably biased by the increased age of patients with a HS pattern compared to patients with an ANoA pattern. Our study therefore does not allow to speculate on the role of antibodies eliciting a HS or nucleolar pattern *in vivo*, neither to predict the development of AID in cancer patients, for which prospective studies are urgently needed.

In conclusion, ANA with a HS pattern were associated with a significantly lower prevalence of neoplastic malignant disease and a decreased relative risk of having cancer compared to other ANA patterns. On the other hand, the nucleolar pattern was associated with a significantly increased relative risk of malignant neoplastic disease. Further studies are needed to investigate which particular antibody/ies against/to NA is responsible for these associations. While anti-DFS70/LEDGF antibodies could be involved in association of the HS pattern with the lower prevalence of cancer, anti-Scl70 and anti-RNAP-III antibodies, the latter being frequently associated with the presence of anti-RNAP-I, are good candidates for the association between ANoA and the presence of cancer, because these antibodies are well known to be associated with cancer and possibly give rise to a nucleolar pattern.

## Data Availability Statement

The datasets generated for this study will not be made publicly available. Despite encrypted crypted, database contain patients numbers that could potentially be used to identify patients. However, database can be provided to the editor to prove the results. Requests to access the datasets should be sent to David Spoerl, david.spoerl@hcuge.ch.

## Author Contributions

DS contributed conception and design of the study. DS and AG organized the database, performed the statistical analysis. AG wrote the first draft of the manuscript. PR-L collected the data and wrote sections of the manuscript. All authors contributed to manuscript revision, read and approved the submitted version.

## Conflict of Interest

The authors declare that the research was conducted in the absence of any commercial or financial relationships that could be construed as a potential conflict of interest.

## Abbreviations

AID: Autoimmune disease
ANA: Antinuclear autoantibody
ANoA: Antinucleolar autoantibody
CENP-A/B: Centromere protein A/B
DFS70: Dense fine speckled antigen
DM: Dermatomyositis
dsDNA: Double-stranded DNA
NA: Nuclear antigens
FITC: Fluorescein isothiocyanate
HEp-2: Human epithelial type 2
ICAP: International consensus on ANA patterns
ICD-10: International classification of diseases, 10th Revision
IIF: Indirect Immunofluorescence
LEDGF: Lens epithelium-derived growth factor
NOR-90: Nucleolus organizer region autoantigen
SLE: Systemic lupus erythematosus
Sm: Smith factor
SjS: Sjögren syndrome
SSc: Systemic sclerosis
PCNA: Proliferating cell nuclear antigen
PM: Polymyositis
PM/Scl: Polymyositis/Scleroderma antigen
RA: Rheumatoid arthritis
RNAP-I: RNA polymerase I
RNAP-III: RNA polymerase III
U3-snoRNP: U3 - small nucleolar ribonucleoprotein particle.
